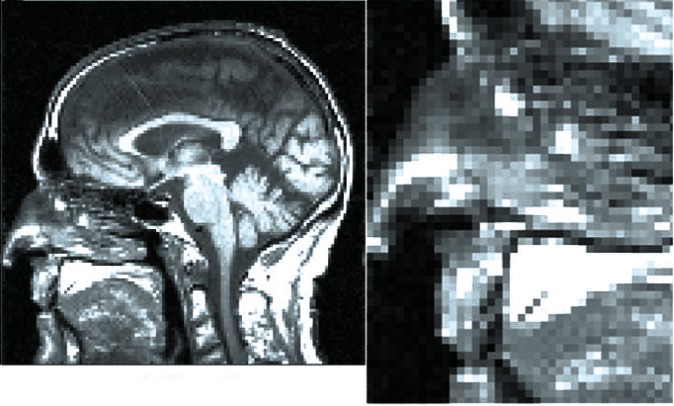# Precision and Accuracy in Scientific Imaging

**DOI:** 10.6028/jres.115.011

**Published:** 2010-06-01

**Authors:** Russell A. Kirsch

**Affiliations:** NIST Emeritus, National Institute of Standards and Technology, Gaithersburg, MD 20899

**Keywords:** digital, image, pixel, scanning, variable

## Abstract

Digital images are commonly used to represent scientific data. Typically, high resolution images with many square pixels are considered to be necessary under the assumption that the increased precision of such images yields increased accuracy to the viewer. We question this assumption by demonstrating improved accuracy in viewing digital images without requiring increased resolution by demonstrating how pixels with variable shapes chosen to best represent an image constitute a significant improvement over the square pixels in enhancing the accuracy of viewing such digital images.

## 1. Introduction

Scientific results are often represented with digital images. The technology of charge coupled devices for which Boyle and Smith won the 2009 Nobel Prize, (1) underscores the importance of the use of this form of representation. Most of those uses follow a tradition, which I began and now recognize as unfortunate, when I introduced the first computer scanned image at the National Bureau of Standards in 1957. (2) Having built SEAC, the first American programmable computer at NBS, it was possible for us to introduce innovations that have survived to the present. SEAC is described at the NIST WEB site: http://museum.nist.gov/panels/seac/SEACOVER.HTM

This first digital image can be downloaded from the NIST Web site: http://www.nist.gov/public_affairs/techbeat/tb2007_0524.htm#image which was released on the 50th anniversary of the first digital image.

The scanner I used divided the scanned image into square pixels. Each pixel had only two possible colors: black or white. The dimensions of the image were 176 × 176 = 30976 such pixels. There were limitations on the quality of the resultant scan. First was the lack of color range.

Over the last half century, developments have improved this such that we now can have digital images with great ranges of color. Other improvements have resulted in scans that may contain many millions of pixels. But one area in which there has been no improvement was in the use of square pixels. This use of square pixels that I started resulted from my lack of awareness of a method used 1500 years previously by the mosaic artists in Ravenna when they chose tesserae carefully by shape and color to best represent their mosaic images. (3) This can be seen at: http://museum.nist.gov/panels/seac/CONSEQ~1.HTM

So it is appropriate a half century later to consider how we might use variable shape pixels to better (or more accurately) represent scientific images. In the examples below we show that the usual assumption that increased precision is accomplished with higher resolution of square pixel images does not necessarily result in the increased accuracy that can be achieved with the use of variable shape pixels each chosen to best represent the image being displayed.

**Fig. 1:**
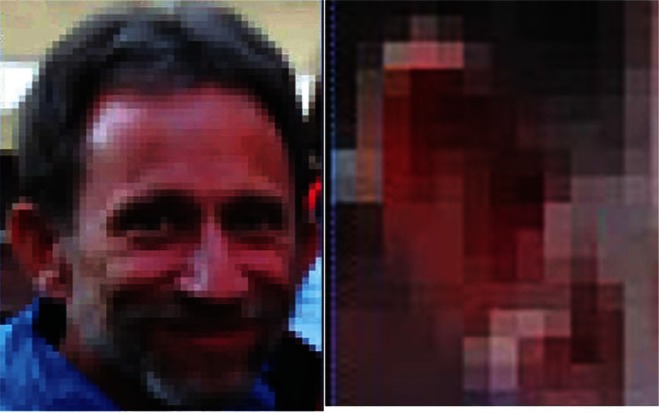


**Fig. 2:**
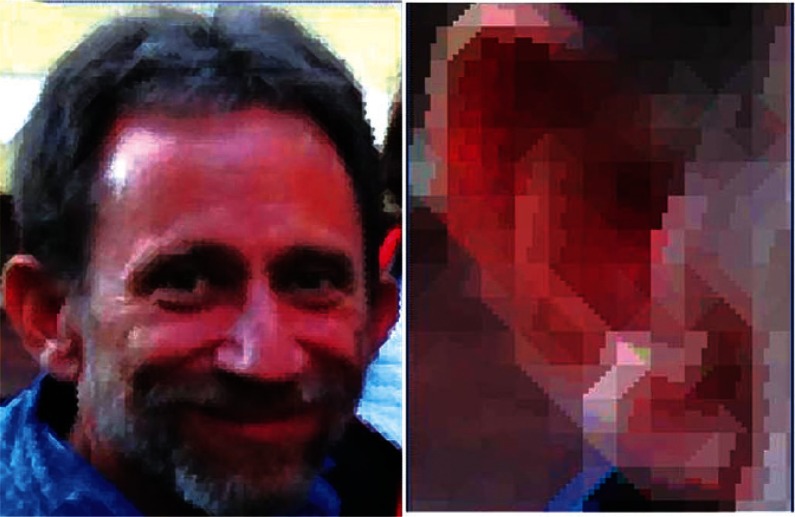


**Fig. 3:**
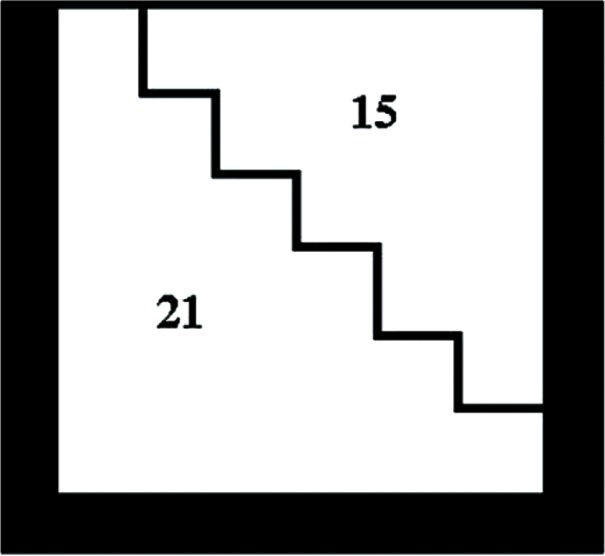


**Fig. 4:**
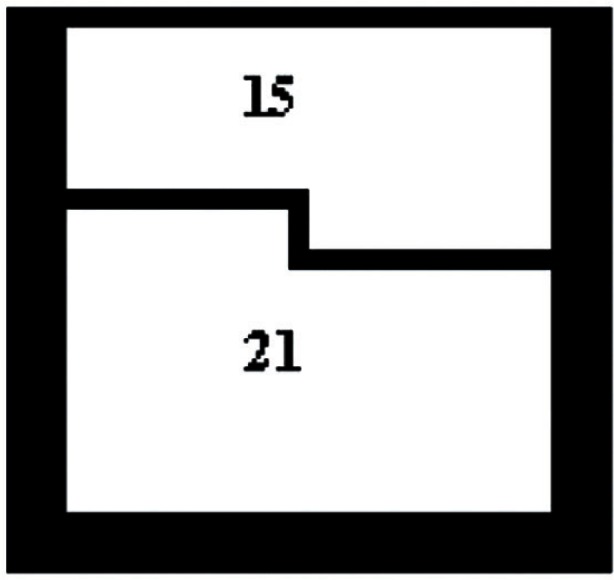


Since the first digital image was of a newborn infant in 1957, I include here two color images of the same 53 year old today using both conventional square pixels in Fig. 1 and variable shape pixels in Fig. 2. A detail of the ear in both images makes the superiority of the variable shape pixel image obvious. The variable shape pixel image was created with a program I wrote in the language MACLISPIX, a programming language written at NIST (formerly NBS) by David Bright in 1995, described at: http://www.nist.gov/lispix/MLxDoc/demo/MASJ/0_intro.html The method used to assign variable shaped pixels is as follows:

The original square pixel image is analyzed through masks of size 6 × 6 pixels. Two such masks built from combinations of square pixels are shown in Figs 3 and 4. Figure 3 consists of two triangles of sizes 21 and 15 pixels. Figure 4 consists of two rectangular shapes also of sizes 21 and 15 pixels. Each of these masks is applied to the original image in four rotations of 90 degrees.

For each of the eight resulting maskings, the average pixel pair values of the masked original image is calculated. Typically the average value will differ for the large and small mask. The masking for which this difference is greatest is chosen and the 36 pixels of the original image are replaced by the two values corresponding to the two shapes. This process is applied to every 6 × 6 pixel array in the original image to produce the variable shaped pixel scan. For an RGB image this process is applied to each of the R, G, and B components of the image and then reconstructed into a single RGB image as shown in Fig. 2.

A comparison of conventional square and new variable shape pixel images: Fig. 1 shows a conventional scanned color image in which all of the red, green, and blue components are square pixels. Fig. 2 shows a color image in which each of the R, G, and B components have been indepentently computed using the pairs of triangular pixels of Fig. 3 and the pairs of rectilinear pixels of Fig. 4. all chosen according to the criterion mentioned above.

We start the comparison by noting that the pairs of triangular pixels consist of one which is 15/16 as large as the corresponding square pixel and the other 21/16 larger. The same is true for the pairs of rectilinear pixels. The component pixels of the variable shape pixel image are as follows:

Red: 1032 triangular pairs and 1728 rectilinear pairs
Green: 1111 triangular pairs and 1649 rectilinear pairs
Blue: 1066 triangular pairs and 1694 rectilinear pairs.

The total number of R,G,B color variable shape pixels is thus 5520.

The square pixel image is of dimension 71 × 94 yielding a total, for comparison, of 6674 pixels. Thus, in an important sense, the square pixel image has more pixels than the variable shape pixel image. But despite this apparently higher resolution, we can see that the conventional square pixel image has more apparent resolution artifacts than the variable shape pixel image. So here we have an example of where apparent increase in precision does not result in increased accuracy.

The storage requirements of the square and variable shape pixel images can be compared. A typical image has pixels with a range of 256 values, each requiring 8 bits. To specify a variable shape pixel image, each pair of pixels requires an additional 3 bits to specify which of the eight shapes and rotations is used. Thus a pair of pixels requires 19 bits compared to the 16 re-quired for a pair of square pixels. Thus the greater accuracy here is achieved at the cost of an additional 18.75 % storage requirement. For different classes of variable shapes this requirement might differ.

**Fig. 5:**
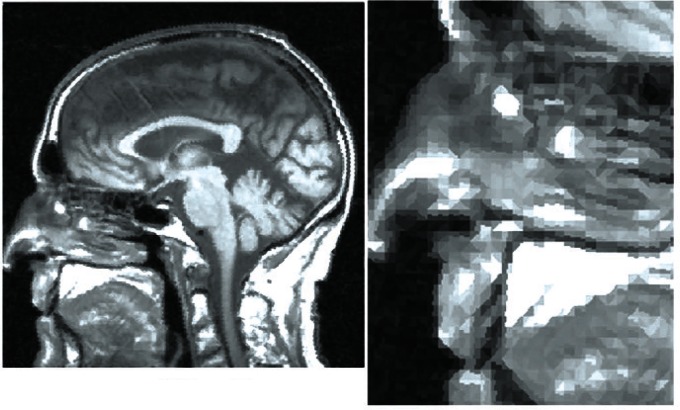


Owing to the ease of viewing human faces the comparison described here is fairly obvious. We wish, however, to illustrate the importance of the use of variable pixels in scientific images. For this purpose, we illustrate an MRI brain scan of myself in the two different methods. Fig. 5 shows the improved variable shape pixel scan and Fig. 6 the conventional square pixel scan. In both these examples there are 13 944 pixels and again we can see the superiority of the variable pixel scan. For clarity, we include an expanded detail of the nose area of both images where, again, the variable and square shapes are evident.

In conclusion we consider that the computer processing of digitally scanned images should emphasize the use of variable shape pixels. This becomes necessary in the absence of technology for performing the original scan in that manner. But insofar as computer processing results in the compression of conventionally scanned images, then the variable shape compression illustrated here should be used.

## Figures and Tables

**Fig. 6:**